# Biological Aging in Breast Cancer Survivors and the Role of Sleep

**DOI:** 10.1093/geroni/igab046.1155

**Published:** 2021-12-17

**Authors:** Judith Carroll

**Affiliations:** University of California, Los Angeles, Los Angeles, California, United States

## Abstract

Although cancer treatments can prolong life, they may lead to long-term changes in physical health and well-being. The lasting symptoms experienced after cancer treatment include greater fatigue, pain, cognitive complaints, and functional decline. Cancer and its related cytotoxic treatments are proposed to directly altering biological aging pathways. Our recent findings support this hypothesis, suggesting that women with breast cancer exposed to therapy have alterations in indicators of biological aging, including elevated DNA damage, reduced telomerase activity, and more rapid epigenetic aging. There was variability in risk for signs of biological aging, and given the high prevalence of sleep problems among breast cancer survivors, we sought to examine whether healthy sleep might be protective. Results suggest that those with good sleep quality had less accelerated biological aging than those with sleep problems. Results point to healthy sleep as a modifiable target to protect women with breast cancer from experiencing biological aging.

